# Posture-related fluctuations of intraocular pressure in healthy children with suspicion of glaucoma

**DOI:** 10.1007/s00417-023-06212-z

**Published:** 2023-08-24

**Authors:** Jan Niklas Lüke, Philip Enders, Alexander Händel, Caroline Gietzelt, Johanna Dietlein, Verena Schöneberger, Alexandra Lappa, Randolf Widder, Thomas S. Dietlein

**Affiliations:** 1 Department of Ophthalmology, Medical Faculty and University Hospital of Cologne, Kerpener Strasse 62, 50937 Cologne, Germany; 2 Department of Ophthalmology, St. Martinus-Krankenhaus Düsseldorf, Gladbacher Str. 26, 40219 Düsseldorf, Germany; 3 Department of Ophthalmology, University Eye Hospital, Rostock, Germany

**Keywords:** Intraocular pressure, Glaucoma, Posture-related, Children, Rebound tonometry

## Abstract

**Purpose:**

Currently, there are no specific data on the circadian course of intraocular pressure (IOP) in children, especially for IOP measurements in the supine position. The study aimed to characterize the diurnal and nocturnal IOP fluctuations in supine and sitting positions in patients less than 18 years of age.

**Methods:**

Seventy-nine eyes of 79 patients under 18 years of age with suspicious optic nerve heads or ocular hypertension could be included in this study. All included patients showed an inconspicuous retinal nerve fiber layer thickness and Bruch’s membrane minimum rim width by coherence tomography. IOP measurements during the 24-h IOP profile were retrospectively evaluated. Measurements were taken at 10:00, 16:00, 20:00, and 23:00 h in the sitting position and at 6:00 h in the morning in the supine position using iCare rebound tonometry on 2 consecutive days.

**Results:**

Thirty-four of 79 children (43.0%) had peak nocturnal IOP values > 25 mmHg. The mean daily IOP was 18.8 ± 5.6 mmHg, and the mean daily fluctuation was 6.1 ± 4.0 mmHg. At 6 am, supine measurements were elevated to 25.1 ± 8.0 mmHg. Extensive fluctuations with values > 40 mmHg in the nocturnal supine measurement occurred in a relevant share of patients (*n* = 5).

**Conclusion:**

There appear to be relevant diurnal and nocturnal IOP fluctuations in healthy children (< 18 years). Nocturnal IOP measurements in supine patients with risk factors for glaucoma may provide important additional information to identify critical patients for further follow-up. 
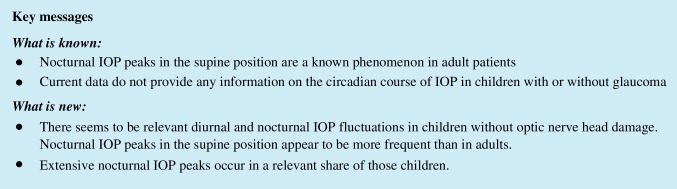

## Introduction

Glaucoma is a chronic disease with multifactorial genesis. The progression of glaucoma leads to a slow increase in visual field loss. Making a confident diagnosis of glaucoma or glaucoma progression can be very difficult. In adult patients, it relies on a functional test, usually standard automated perimetry, and morphologic imaging. Techniques such as optic disc optical coherence tomography allow comparison of baseline and follow-up examinations and can quantify structural damage over time. Both of these examinations can be very difficult for children because of their limited ability to cooperate. Nevertheless, optical coherence tomography can be a helpful tool to differentiate glaucoma from suspicious optic discs such as macrodiscs.

Elevated intraocular pressure (IOP) is considered a major risk factor for the onset and progression of glaucoma. Likewise, IOP is the most accessible risk factor for a medical or surgical therapeutic approach to prevent progression. Although elevated IOP is considered a major risk factor, other factors such as blood pressure and ocular perfusion pressure also appear to play an important role in disease progression [1, 2].

There are various methods for monitoring IOP. In addition to Goldmann applanation tonometry, which is still considered the gold standard, rebound tonometry is a method that has become increasingly established in clinical practice. The advantage of rebound tonometry is that it can be performed easily and without conjunctival anesthesia in different body positions.

Variations in intraocular pressure are known to occur both in different body positions and over a period. In the literature, a distinction is commonly made between short-term and long-term IOP fluctuations, although there is no agreed definition for either term. Frequently, short-term fluctuations refer to the difference between maximum and minimum values within 24 h; more rarely, this term refers to the standard deviation within this period [3, 4]. In general, IOP in adult patients usually peaks in the early morning [5]. Long-term variations usually refer to differences between measurements on different days that may be weeks or even months apart [6].

In the supine position, IOP is elevated compared to the sitting position [7]. Postural fluctuations of intraocular pressure in humans have been known for a long time. This phenomenon exists physiologically but seems to be increased in glaucoma patients [7]. Based on this fact, it seems possible that the development of glaucoma as well as the progression of the disease is related to increased postural IOP variability, although this is controversial [8–10]. The exact pathogenesis of this mechanism is still not fully understood. Furthermore, it is unclear whether the increased fluctuations develop already in childhood and whether increased IOP fluctuations can lead to glaucomatous damage of the optic nerve already in childhood.

Our study aimed to investigate the prevalence and magnitude of postural IOP peaks and diurnal IOP fluctuations in children with suspicion of glaucoma.

## Materials and methods

### Study design

The medical records of all patients referred to our tertiary glaucoma center for 24-h IOP profiling between 2015 and 2021 were retrospectively reviewed to meet all inclusion and no exclusion criteria. Inclusion criteria were age up to 18 years, macrodiscs, or optic disc anomalies. When perimetry could be performed, no significant and reproducible defects were detectable. Because of young age, reliable visual field analysis was not feasible in many cases.

Exclusion criteria included a diagnosis of glaucoma and nonglaucomatous ocular diseases that could affect correct tonometry or optic disc morphology (e.g., superficial keratopathy or prematurity) were reasons for exclusion. Other exclusion criteria included a diagnosis of any syndrome, high refractive error (more than +5/−5dpt hyperopia or myopia), high astigmatism (more than −3dpt), history of refractive surgery, or intraocular surgery. In contrast, amblyopia or strabismus was not specified as a reason for exclusion. Patients who already had a definite glaucoma diagnosis due to specific changes in OCT of the optic disc and specific perimetric defects and patients who were already treated with antiglaucomatous drugs were excluded.

The records of 7488 patients admitted to the hospital for measurement of the 24-h IOP profile during the above period were reviewed. Of these, 271 patients were eligible based on age. One hundred ninety-two patients had to be excluded based on the above criteria.

### IOP measurement

The IOP measurements for the right eye were considered for the calculation. Measurements were taken at 10:00, 16:00, 20:00, and 23:00 h in the sitting position and at 6:00 h in the supine position using iCare TA01i rebound tonometry (Tiolat Oy, Helsinki, Finland). All examiners paid attention to the correct position of the tonometer with a distance of 4–8 mm from the cornea, as prescribed by the manufacturer. Patients were instructed to lie flat in the supine position for at least 3 h before the measurement at 6 am. The device automatically calculated an average value from 6 consecutive individual measurements [11]. Good comparability of rebound tonometry with Perkins applanation tonometry was found both in supine adults and in children under anesthesia [11, 12].

The diurnal fluctuation was determined as the difference between the minimum and maximum IOP during the day (10:00–23:00). The posture-dependent divergence was calculated as the deviation of the mean diurnal IOP from the nocturnal measurements (6:00).

### Ethical approval and statistical analysis

According to regulations of the professional code for Physicians, all tenets of the Declaration of Helsinki have been regarded.

Descriptive statistics were performed to obtain a more accurate characterization of the study group. Normal distribution was tested using the D’Agostino-Pearson normality test. Because IOP values were not normally distributed, the Mann-Whitney test was performed to assess significance. Values were expressed as mean ± standard deviation of the mean (SD). As a nonparametric comparison, the Spearman rank correlation test was used to compare parameters in the case of parameters that were not normally distributed. Poor, moderate, and excellent reproducibility were considered as values of *r* < 0.4, 0.4 < *r* < 0.75, and *r* > 0.75, respectively. Statistical significance was set at *p* < 0.05. Other high significance levels were set at *p* < 0.01 and *p* < 0.001. All analyses and data presentations were performed using Excel (Microsoft Office Excel 2016, California, USA), SPSS v. 22 (IBM Chicago, Illinois, USA), and GraphPad software (GraphPad Prism 7, Inc, La Jolla, USA).

## Results

Seventy-nine eyes of 79 patients (right eye only) with a mean age of 11.4 ± 3.7 years (range: 5 to 17 years) were included in the analysis. The best-corrected visual acuity was 0 ± 0.1 logMAR on average. The mean corneal thickness was 569 ± 35.4 μm. In the first group with a suspicious optic disc (*n* = 69), the mean global Bruch’s membrane opening minimum rim width was 277.3 ± 55.1 μm, while the values were higher in 10 patients with ocular hypertension (355.9 ± 65.5 μm). The mean peripapillary thickness of the retinal nerve fiber layer was similar in both groups (suspect optic disc 94.7 ± 11.7 μm/ocular hypertension 94.4 ± 12.4 μm). BMO area was not significantly different in the group with suspicious ONH (2.3 ± 0.5 mm^2^) compared with the ocular hypertension group (2.0 ± 0.5 mm^2^) (Table 1).

**Table 1:** Epidemiological data of the study cohort

	Study eyes (*n* = 79)	Ocular hypertension (*n* = 10)	Suspect optic disc (*n* = 69)
Age (years)			
Mean ± SD	11.4 ± 3.7	12.1 ± 3.8	11.3 ± 3.7
Range	5–17	5–16	5–17
BCVA at baseline in logMAR			
Mean ± SD	0.0 ± 0.1	0 ± 0.1	0 ± 0.1
Cup-disc-ratio			
Mean ± SD	0.6 ± 0.2	0.3 ± 0.1	0.6 ± 0.1
Pachymetry (in μm)			
Mean ± SD	569 ± 35.4	578.1 ± 24.9	567.7 ± 36.7
OCT parameters			
Mean Global BMO-MRW in μm	287.2 ± 61.8	355.9 ± 65.5	277.3 ± 55.1
Mean BMO area in mm^2^	2.3 ± 0.5	2.0 ± 0.5	2.3 ± 0.5
Mean Global RNFL thickness	94.7 ± 11.7	94.4 ± 12.4	94.7 ± 11.7

The mean daily IOP values during the first 2 consecutive days of 24-h measurement were 20.8 ± 5.8 mmHg in the ocular hypertension group and 18.5 ± 5.6 mmHg in the suspected optic disc group. Mean daily fluctuations were 6.1 ± 4.0 mmHg and 7.7 ± 4.4 mmHg (second day). The position-dependent divergence was 7.3 ± 6.1 mmHg (6.0 ± 4.1mmHg on the second day) (Tables 2, 3, and 4).

**Table 2:** IOP values during the 48 h measurement

*n* = 79	IOP 10 h	IOP 16 h	IOP 20 h	IOP 23 h	IOP 6h (lying position)	Fluctuation (delta min./max.)	Posture/nocturnal divergence (delta 6 h/mean diurnal IOP)
Mean IOP (day 1)	19.0 ± 4.0	19.2 ± 6.3	18.5 ± 6.5	18.5 ± 6.4	25.1 ± 8.0	6.1 ± 4.0	7.3 ± 6.1
Mean IOP (day 2)	20.0 ± 4.7	20.6 ± 5.1	18.3 ± 4.9	19.9 ± 7.1	24.3 ± 6.9	7.7 ± 4.4	6.0 ± 4.1

**Table 3:** IOP values during the 48 h measurement of patients with ocular hypertension

Ocular Hypertension *n* = 10	IOP 10 h	IOP 16 h	IOP 20 h	IOP 23 h	IOP 6 h (lying position)	Diurnal fluctuation (delta min./max.)	Posture-related divergence (delta 6 h/mean diurnal IOP)
Mean IOP (day 1)	13.1 ± 8.8	20.4 ± 8.4	18.8 ± 9.3	17.3 ± 8.9	25.9 ± 3.3	7.2 ± 5.6	5.0 ± 4.2
Mean IOP (day 2)	19.1 ± 7.3	20.8 ± 9.9	17.0 ± 9.3	21.2 ± 13.9	24.8 ± 4.8	8.3 ± 2.7	3.2 ± 2.5

**Table 4:** IOP values during the 48 h measurement of patients with suspicious optic discs

Suspect optic disc (*n* = 69)	IOP 10 h	IOP 16 h	IOP 20 h	IOP 23 h	IOP 6 h (lying position)	Fluctuation (delta min./max.)	Posture/nocturnal divergence (delta 6 h/mean diurnal IOP)
Mean IOP (day 1)	18.5 ± 5.3	18.5 ± 6.7	17.9 ± 6.7	18.1 ± 6.7	25.0 ± 8.5	6.0 ± 3.8	7.7 ± 6.2
Mean IOP (day 2)	19.5 ± 5.4	19.8 ± 5.7	17.7 ± 5.3	18.9 ± 6.9	24.2 ± 7.1	7.6 ± 4.5	6.4 ± 4.1

The measurement in the supine position differed significantly from the respective daily mean within each group (*p* < 0.001).

No significant correlation could be found between the age of the patients and the daily mean intraocular pressure or the level of intraocular pressure when measured in the supine position.

A significant moderate correlation was found between the mean diurnal IOP and the pachymetry values (*p* < 0.001; *r* = 0.623) as well as a significant weak correlation between the absolute IOP value in the supine position and the pachymetry values (*p* < 0.05; *r* = 0.291).

To assess the reproducibility of the measurements as a function of the time of day, the IOP values of all 79 patients were correlated with the measurements of the following day. When comparing the exact measurement points of 2 consecutive days, a moderately significant correlation was found (*r* between 0.325 and 0.745; *p* < 0.05). Nocturnal divergence correlated significantly with the values of consecutive days (*r* = 0.317; *p* < 0.05). No significant correlation was found between the level of nocturnal fluctuation over the 2 consecutive days (*r* = 0.07; *p* = 0.589).

**Fig. 1:**
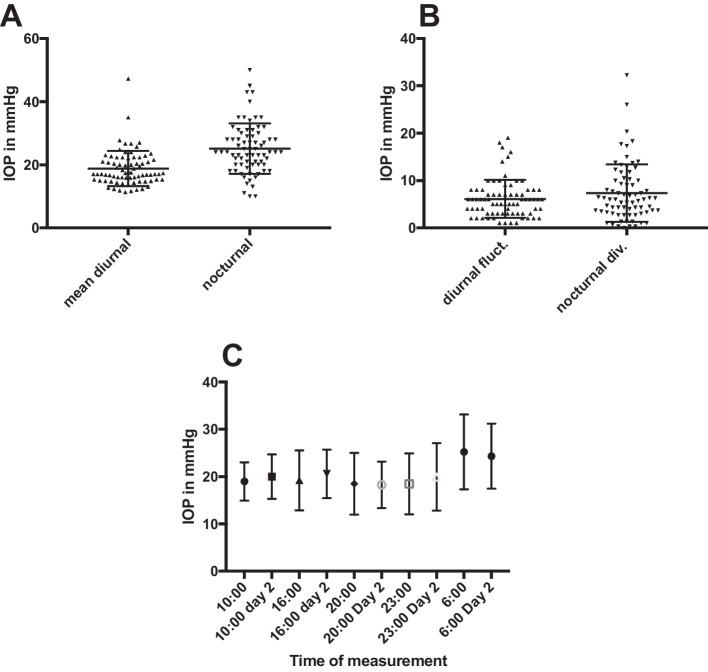
Illustration of distribution of IOP values and fluctuation. **A** Mean diurnal IOP values of each patients compared to the nocturnal measurements in a spinal position is illustrated as a single dot. The columns present mean values ± standard deviation (SD). **B** Maximum of diurnal fluctuation as well as the nocturnal divergence from the diurnal mean value of each patient is presented for both groups with dots. The columns present mean values of the groups with SD. **C** Average values of the measurements at the specific times of day compared with the measurements of the consecutive days as well as the standard deviation are shown as bars

The total number of all patients with peak IOP values (> 25 mmHg) in the supine position at 6 am was 34 (43.0%). Five of ten children with ocular hypertension had peak IOP values (> 25 mmHg) in the supine position. One patient was found to have wide variations up to 50 mmHg when measured in the supine position. In four patients, values between 40 and 49 mmHg were measured. In *n* = 13 cases, IOP was elevated to values between 30 and 39 mmHg in the supine position (Fig. 1).

Comparing the mean pachymetry of children with peak IOP values below 25 mmHg (558 μm) with that of children with values above 25 mmHg (578 μm), a significant difference was found (*p* < 0.05).

To assess possible incipient glaucomatous damage due to extended nocturnal IOP peaks, we calculated the correlation between IOP measured in the supine position and global RNFL thickness as well as RNFL thickness in each sector (temporal inferior, temporal, temporal superior, nasal inferior, nasal superior). No significant correlation was found. Furthermore, no significant correlation was found between the daily mean IOP and RNFL thickness in each sector.

## Discussion

It has been suggested that large fluctuations in intraocular pressure during the day and night are related to the progression of visual field loss in adult glaucoma patients [13].

Most studies investigating postural and diurnal variations in intraocular pressure exclude subjects younger than 18 years [14, 15]. In a previous retrospective study from Germany, 121 glaucoma patients hospitalized for measurement of circadian IOP had a mean age of 66 ± 15 years [16].

In clinical practice, the capacity for 24-h IOP measurement is usually very limited. The indication for inpatient admission of children for 24-h pressure measurement is only exceptional, resulting in a lack of information about the circadian rhythmicity of IOP in children.

Most studies in adult healthy individuals or medically controlled glaucoma patients measure mean IOP values in the range of 13–16 mmHg, whereas patients with ocular hypertension have higher values between 20 and 25 mmHg during day-night hours [14, 16, 17]. The mean daily values of patients in this study hospitalized for optic disc anomalies or ocular hypertension can be classified above the upper limit of this normal range, with measurements in the supine position being even higher.

In an adult cohort of 44 patients (88 eyes, 68.7 ± 10.8) with ocular hypertension or primary open-angle glaucoma (POAG), stable reproducibility of mean intraocular pressure, minimum and maximum intraocular pressure, and intraocular pressure fluctuation were described in an individual when comparing 2 consecutive days of daily intraocular pressure measurements [18].

Mean daily variations in the intraocular pressure range from 3 to 6 mmHg, with lower values in healthy adult subjects or patients with suspicious optic discs than in patients with ocular hypertension or glaucoma [14, 16, 19]. In this study, we found that diurnal fluctuations in children can be classified around the upper limit of these ranges. Some authors postulate that the magnitude of IOP fluctuations can vary widely interindividual, but that the pattern of IOP fluctuations in the same individual is relatively stable [19]. When comparing the time-dependent measurements and fluctuation on 2 consecutive days in our cohort, a significant correlation (*p* < 0.05) was found for the parameters (10:00, 16:00, 20:00, 23:00, 6:00 values, postural divergence). In contrast, the minimum/maximum diurnal fluctuation was different on the 2 consecutive days. Thus, the latter hypothesis seems to be applicable to younger patients except for the magnitude of fluctuation during the day. In adults, differences in intraocular pressure of > 2 mmHg between days 1 and 2 were found in a substantial number of cases [20]. Consequently, the 48-h hospital stay of patients for measurement of IOP seems to be an appropriate period to collect information about individual circadian IOP rhythmicity.

Pronounced IOP peaks in the supine position appear to be difficult to control in adult patients through drug therapy or non-filtering glaucoma surgery [21]. In cases of significant glaucoma progression, only filtering glaucoma surgery seems to be effective in significantly reducing postural IOP variation in adult patients [21]. The reason for the advantage of the filter method is thought to be that the increased venous resistance of the episcleral veins in the supine position is bypassed when the aqueous humor is drained into the subconjunctival space [22].

Konstas et al. and Fogagnolo et al. detected peak intraocular pressure outside regular office hours in 30–45% of their glaucoma patients during 24-h intraocular pressure monitoring [19, 23]. Hughes et al. found that in 14% of their patients with primary open-angle or normal-tension glaucoma, peak IOP during the night was at least 12 mmHg higher than peak IOP during office hours [24]. Although mean intraocular pressure was not significantly different during office hours and 24-h measurements, peak intraocular pressure values were significantly lower during office hours compared with circadian measurements. Similarly, Nakakura and coworkers showed that the fluctuations during office hours (2.7 mmHg) were significantly lower than the fluctuations during 24-h measurements (6.7 mmHg). This observation is consistent with the mean value of the 24-h IOP fluctuation of the children examined by our study group (7.3 ± 6.1 mmHg).

Interestingly, in the present study, no correlation was found between the mean diurnal IOP and the RNFL thickness of the different sectors, nor between the absolute supine position IOP measurements and the RNFL thickness. The extent to which higher short-term IOP fluctuations lead to glaucoma progression is still controversial [25]. It can be discussed whether the susceptibility to glaucomatous damage due to extensive IOP fluctuations in supine position is reduced in childhood. This assumption could be based on the higher elastic properties of the neuronal tissue at a young age [26]. To further develop this hypothesis, a longitudinal study with OCT progression data is needed. On the other hand, these results could possibly be attributed to the heterogeneity of the patients in this study (ocular hypertension and suspicious ONH).

In both groups, the pachymetry values showed slightly increased to similar values around 570 μm. A significant correlation was found between the mean diurnal IOP and the pachymetry values as well as between the absolute IOP value in the supine position and the pachymetry values. When interpreting the IOP values with slightly increased pachymetry values, it must be considered that the values can be overestimated, especially in the case of IOP measurements with rebound tonometry. In a study with mean pachymetry of 558 ± 36 μm, values were approximately 2 mmHg higher with rebound tonometry than with Goldmann applanation tonometry in children [27]. It can therefore be assumed that the partly substantially increased IOP values are not solely attributable to rebound tonometry. Furthermore, it must be taken into account that, especially in children, the measurement with Goldmann applanation tonometry is often not feasible; failure rates of up to 26% have been described [27].

Limitations of this study include the retrospective study design and the lack of a healthy control group. Also, the fact that the indication for 24-h IOP profiling was based on different diagnostic criteria (ocular hypertension versus suspicious optic disc) may lead to a heterogeneous cohort and thus bias. However, we think that the large cohort of patients that could be included in this study reduces this effect.

In addition, the lack of differences in IOP fluctuation and absolute IOP values between the 2 groups is probably due to the small number of cases in the ocular hypertension group.

On the basis of the IOP values collected in our study, it can be suggested that postural fluctuations and diurnal pressure fluctuations seem to be higher in young patients (< 18 years). Thirty-four of 79 patients had peak IOP values of > 25 mmHg at night, which might lead to the conclusion that these patients should be examined more frequently and carefully. Despite the particularly pronounced IOP spikes, coherence tomography did not detect any incipient damage or difference between the children with and without IOP spikes, which may indicate a low susceptibility of the juvenile optic disc to posture-dependent IOP fluctuations.

However, further prospective studies investigating the occurrence or long-term course of glaucoma due to postural IOP fluctuations at a young age are needed.

## Abbreviations

*IOP*: intraocular pressure
*BCVA*: best-corrected visual acuity
*SD*: standard deviation of mean
*ONH*: optic nerve head
*BMO*: Bruch’s membrane opening
*RNFL*: retinal nerve fiber thickness
